# Using equivalence class counts for fast and accurate testing of differential transcript usage

**DOI:** 10.12688/f1000research.18276.2

**Published:** 2019-04-29

**Authors:** Marek Cmero, Nadia M. Davidson, Alicia Oshlack

**Affiliations:** 1 Murdoch Childrens Research Institute, Parkville, Victoria, 3052, Australia; 2 School of BioScience, University of Melbourne, Parkville, Victoria, Australia

**Keywords:** RNA-seq, differential transcript usage, equivalence class, transcript compatibility class, pseudo-alignment, DEXSeq, Salmon, Kallisto

## Abstract

**Background:** RNA sequencing has enabled high-throughput and fine-grained quantitative analyses of the transcriptome. While differential gene expression is the most widely used application of this technology, RNA-seq data also has the resolution to infer differential transcript usage (DTU), which can elucidate the role of different transcript isoforms between experimental conditions, cell types or tissues. DTU has typically been inferred from exon-count data, which has issues with assigning reads unambiguously to counting bins, and requires alignment of reads to the genome. Recently, approaches have emerged that use transcript quantification estimates directly for DTU. Transcript counts can be inferred from 'pseudo' or lightweight aligners, which are significantly faster than traditional genome alignment. However, recent evaluations show lower sensitivity in DTU analysis compared to exon-level analysis. Transcript abundances are estimated from equivalence classes (ECs), which determine the transcripts that any given read is compatible with. Recent work has proposed performing a variety of RNA-seq analysis directly on equivalence class counts (ECCs).

**Methods:** Here we demonstrate that ECCs can be used effectively with existing count-based methods for detecting DTU. We evaluate this approach on simulated human and drosophila data, as well as on a real dataset through subset testing.

**Results:** We find that ECCs have similar sensitivity and false discovery rates as exon-level counts but can be generated in a fraction of the time through the use of pseudo-aligners.

**Conclusions:** We posit that equivalence class read counts are a natural unit on which to perform differential transcript usage analysis.

## Introduction

RNA sequencing with short-read sequencing technologies (RNA-seq) has been used for over a decade for exploring the transcriptome. While differential gene expression is one of the most widely used applications of this data, significantly higher resolution can be achieved by using the data to explore the multiple transcripts expressed from each gene locus. In particular, it has been shown that each gene can have multiple isoforms, sometimes with distinct functions, and the dominant transcript can be different across samples ^1^. Therefore, one important analysis task is to look for differential transcript usage (DTU) between samples.

Several approaches already exist to characterise DTU. Transcript-assembly based approaches (such as *cufflinks/cuffdiff*) ^2^ deconvolve transcript read distributions and test differences in inferred transcript abundances. Other methods consider reads supporting particular isoforms or junctions (such as MISO ^3^ or *leafCutter* ^4^). Alternatively, DTU can be inferred through differences in exon usage, where the proportions of RNA-Seq reads aligning to each exon change relative to each other between biological groups. Anders *et al.* ^5^ showed that exon read counts could be used to test for differential exon usage with a generalized linear model that accounts for biological variability. However, counting fragments across exons is not ideal because many fragments will align across multiple exons, making their assignment to an individual exon ambiguous. Moreover, individual exons often need to be partitioned into multiple disjoint counting bins when exon lengths differ between transcripts. Genomes of complex organisms typically contain more exons per gene than transcripts per gene. Supplementary Table 2 shows the human reference example, with an average of 3.5 transcripts per gene and 11.9 exons per gene. Spreading information over a larger number of bins, such as exons that are always transcribed together, results in lower power in statistical tests for testing for differences between samples.

**Figure 1.**
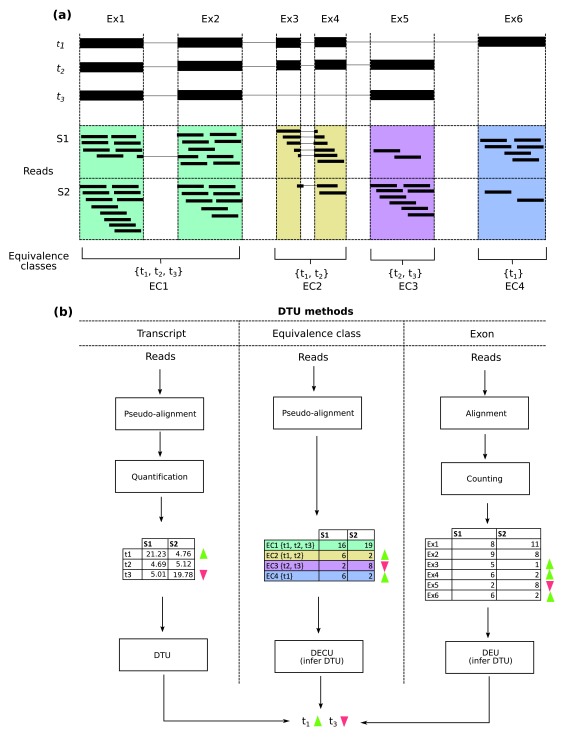
The use of equivalence classes for detecting differential transcript usage (DTU) in a hypothetical gene. The example shows a gene consisting of six exons (Ex1-6) and three transcripts (t _1-3_) resulting in four equivalence classes (EC1-4). t _1_ is predominantly expressed in condition 1 (S1), whereas t _3_ is predominantly expressed in condition 2 (S2). The DTU is evident as a change in the relative counts for EC2, EC3 and EC4 between conditions. The pipelines for the three alternative methods for detecting DTU are shown: quantification of transcript expression followed by DTU testing, assignment of read counts to equivalence classes followed by testing of equivalence class counts (DECU) and assignment of read counts to exons followed by differential exon counts (DEU). Genes that are detected to have DECU or DEU are inferred to have DTU. The transcript quantification table in the left-most column is example data only, and is not based on real inference.

An alternative to using exon counts for testing DTU is to perform tests directly on estimated transcript abundances ^6^. Recently, fast and accurate methods for quantifying gene expression at the transcript level have been developed ^7, 8^. These methods use transcript annotations that include multiple known transcript sequences for each gene as a reference for the alignment. The expression levels of individual transcripts can be estimated from pseudo-aligned reads that are compatible with transcripts associated with a specific gene ^9^. Transcript abundance estimates can be used as an alternative starting measure for DTU testing ( Figure 1b), which has been shown to perform comparably with state-of-the-art methods ^6^. In addition, pseudo-alignment is significantly faster than methods that map to a genome. However, in the most comprehensive comparison using simulated data, exon-count based methods were shown to have slightly better performance compared with methods that first estimate transcript abundances ^6^.

Conceptually, quantification by lightweight or ‘pseudo’ alignment begins by using a transcript annotation as a reference and then assigns each read as ‘compatible’ with one or more transcripts that are a close alignment to the read ^7^. Because different transcripts of the same gene share large amounts of sequence, many reads are compatible with several transcripts. Reads are therefore assigned to an equivalence class, or transcript compatibility class, which reflects the combination of transcripts compatible with the read sequence ( Figure 1). For the purposes of this work, we consider an equivalence class to be defined as in Bray *et al.* ^7^, i.e. any fragments that are pseudo-aligned to the same set of transcripts are considered to be part of the same equivalence class. Figure 1a shows a toy example of a gene with three different transcripts. Depending on its sequence, a read can align to all three transcripts, only two of the transcripts or just one transcript. These different combinations result in four observed equivalence classes, containing read counts, for this gene (the ECs containing uniquely t2 and uniquely t3 are not reported as we do not observe reads for them).

Recently, equivalence class counts have been used for clustering single-cells ^10, 11^ and Yi and colleagues have recently introduced direct differential testing on equivalence classes in a method to identify genes that display differential transcript expression phenomena such as cancellation (isoform switching), domination (high abundance isoform(s) that mask transcript-level differences) and collapsing (multiple transcripts exhibiting small changes in the same direction) ^9^. This methodology utilises aggregation of p-values to identify differential transcript expression. Isoform switching, however, cannot be distinguished using this method if gene expression is also changing between conditions. Here we focus on detecting differences in isoform expression irrespective of gene expression differences, using methods originally designed for testing exon read counts. We evaluate the appropriateness of equivalence class read counts as an alternative choice for quantification compared to exon- and transcript-level quantification. We propose that DTU can be more accurately detected using equivalence class counts directly, rather than using these counts to first estimate individual transcript abundances before performing DTU. Soneson *et al.* applied a conceptually similar method with MISO ^3^ by defining counting bins as combinations isoforms and counting according to isoform compatibility ^6^. In our scenario, count-based DTU testing procedures such as DEXSeq are applied directly to equivalence class counts generated from fast lightweight aligners, such as Salmon and Kallisto. DTU testing on equivalence class counts is not only fast but also bypasses inherent uncertainty in directly estimating transcript abundances before statistical testing.

We evaluate the performance of DTU testing on equivalence class read counts using real and simulated data, and show that the approach yields higher sensitivity and lower false discovery rates than estimating counts from transcript abundances, and performs faster with accuracies similar or better than counting across exons.

## Results

The method we propose is to first perform alignment with a lightweight aligner and extract equivalence class (EC or transcript compatibility) counts. These ECCs are assigned to genes using the annotation of the transcripts matching to the EC. Next, each gene is tested for DTU between conditions using a count based statistical testing method where exon counts are replaced with EC counts ( Figure 1b). Significant genes can then be interpreted to have a difference between the relative abundance of transcripts of that gene between conditional groups. In evaluating the EC approach, we used Salmon for pseudo-alignment and DEXSeq for differential testing. We then compared DTU results against the alternative quantification and counting approaches, also using DEXSeq for testing (see Methods). It should be noted that we are not attempting to evaluate the statistical testing method (DEXSeq) in relation to other methods, as this has been done previously in several papers ^6, 12, 13^.

We evaluated performance on both simulated and real datasets, using simulated data from human and drosophila from Soneson *et al.* ^6^, simulated human data from Love *et al.* ^13^ and biological data from Bottomly *et al.* ^14^. Each of the Soneson datasets consists of two sample groups, each with three replicates, where 1000 genes were randomly selected to have DTU such that the expression levels of the two most abundant transcripts were switched. The Love *et al.* data contains genes defined with differential transcript expression and DTU across two groups with 12 replicates each. The Bottomly dataset contains 10 and 11 replicates each from two mouse strains that were used to call truth and then were subsampled to three replicates in the testing scenarios.

### Fewer equivalence classes are expressed than exons

**Figure 2.**
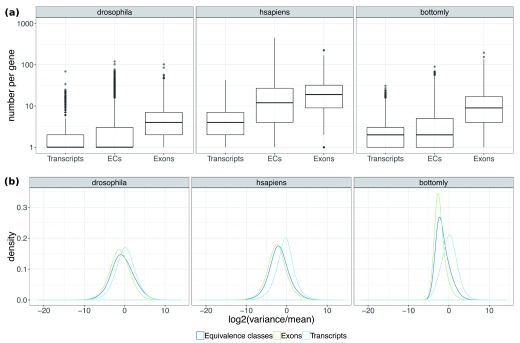
The number of counting bins and variance between replicates. ( **a**) The number of transcripts, equivalence classes and exons per gene, where each feature has at least one associated read. ( **b**) The density of the log _2_ of the variance of counts over the mean for each feature (calculated per condition).

The number of counting bins used for DTU detection has an impact on sensitivity. More bins leads to lower average counts per bin and therefore lower statistical power per bin and more multiple testing correction. We therefore examined the number of ECs, transcripts and exons present in each dataset. Although the theoretical number of ECs from a set of transcripts can be calculated from the annotation and has the potential to be large, not all combinations of transcripts exist or are expressed. The number of equivalence classes calculated from pseudo-alignment depends on the experimental data as only ECs with reads assigned to them are reported. We compared the number of transcripts and exon bins in the three datasets (with at least one read) to the number of reported ECs. In both the simulated human and drosophila datasets, as well as in the Bottomly mouse data, the number of ECs is greater than the number of transcripts, but substantially fewer than the number of exons, indicating that there might be more power for testing DTU using ECCs, compared to exon counts ( Figure 2a).

### Equivalence class replicates have low variance

In addition, we found that the variability of counts across replicates calculated from ECs was lower than that from estimated transcript abundances across all three data sets ( Figure 2b). Count variability of ECs was on average closer to the exon count variability distribution than transcript count variability. For instance, the Bottomly data had an average log _2_ variance to mean ratio of -2.249 and -1.519 in exons and ECs respectively, compared to 0.115 in transcripts. The simulated data followed a similar pattern. Supplementary Figure 1 ^15^ shows the dispersion-mean trends, again demonstrating lower dispersion in ECs compared to transcript abundance estimates. We hypothesise that the greater dispersion observed for transcript data arises from the abundance estimation step used by pseudo-aligners to infer transcript counts. Due to the lower dispersion, we anticipate that analysis of ECCs yield greater power for DTU compared to transcript abundance estimates.

### Performance of equivalence classes for DTU detection

**Figure 3.**
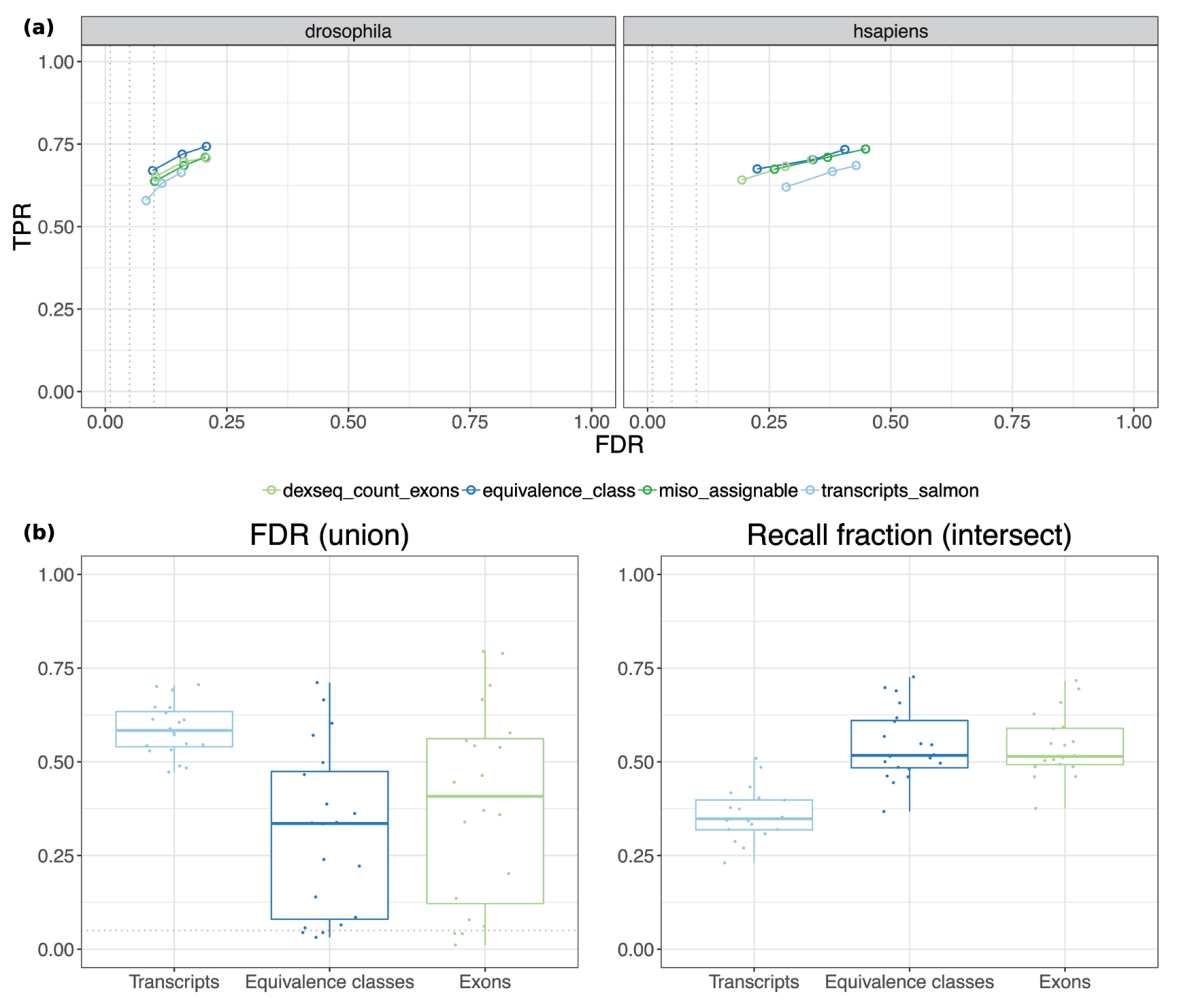
The performance of the equivalence class method for differential transcript usage. ( **a**) The equivalence class method compared to other state-of-the-art methods on simulated data described in Soneson *et al.* ^6^. The circles show the observed true positive rate (TPR) versus false discovery rate (FDR) at three nominal FDR cut-offs (0.01, 0.05 and 0.1) for each method. The dotted lines indicate the FDR at the nominal cut-off values of 0.01, 0.05 and 0,1 (i.e. if the FDR is adequately controlled, the three circles should match up with these lines). ( **b**) The ability of the equivalence class, transcript and exon-based methods to recreate the results of a full comparison (10 vs. 11) of the Bottomly data, using only a (randomly selected) subset of samples (3 vs. 3) across 20 iterations using FDR < 0.05 (FDR = 0.05 is indicated by the dotted line). The union of all genes called as significant across all three methods is used to calculate the FDR, and the intersect (genes called by all three methods) is used for the TPR. Full results (union, intersect and each method’s individual truth set) are shown in Supplementary Figure 3, which also shows lines connecting the results from each iteration.

Several methods were previously tested on the simulated data from Soneson *et al.* ^6^; DEXSeq’s default counting pipeline and featureCounts were shown to perform best. We recalculated exon counts using DEXSeq’s counting pipeline (as recommended by Soneson *et al.*, we excluded region of genes that overlapped on the same strand in the input annotation) and ran Salmon ^8^ to obtain both transcript abundance estimates and equivalence class counts. All other comparison results were obtained from Soneson *et al.* ^6^. We also included MISO’s results as the method was implemented in a conceptually-similar way to our proposed EC method. For the simulated datasets, we found that ECs had the highest sensitivity in both the drosophila and human datasets ( Figure 3a) with a TPR of 0.743 and 0.734 respectively at FDR < 0.1. However, ECs also had a slightly higher FDR in the human data than the exon-counting method.

We next tested the performance of the EC method on a biological dataset from Bottomly *et al.* We tested the complete RNA-seq dataset (10 vs. 11) for DTU using DEXseq on counts generated from transcript abundance estimates, exons and ECs. To calculate the FDR, we considered the set of 'true' DTU genes to be the union of all genes called significant (FDR < 0.05) across the three methods. To calculate the TPR, the intersect of genes called by all methods was used. Supplementary Figure 2 ^15^ shows the number of significant genes and overlap between all three methods. Exons called the highest number of genes with significant DTU (748 genes, compared with 675 significant genes called by ECs). In contrast, transcripts called the fewest number of significant genes (391). Similar to the FDR experiments described in Pimentel *et al.* ^16^, we randomly selected three samples per condition and performed DTU using all three methods and repeated this for 20 iterations. Figure 3b shows the results. ECC-based testing performed the best, with a mean FDR of 0.305 across all iterations (compared to a mean FDR of 0.569 and 0.373 for the transcript and exon-based methods respectively). The mean recall fraction was also slightly higher for ECs at 0.544, compared to exons at 0.539 and 0.36 for the transcript-based method. Results for all three combinations of the ‘truth gene’ sets (union, intersect and individual) are shown in Supplementary Figure 3 ^15^. The ECC-based method had consistently lower number of false positives, which is also illustrated by the rank-order plot (Supplementary Figure 4 ^15^), showing the number of false positives present in the top 500 FDR-ranked genes. The range of FDR results across different subsets of the data was generally higher for ECCs and transcript counts, which may be the result of substructure in the data. However, FDR was similar to exon-counts in each random selection and consistently lower than in transcripts (except in one iteration; see Supplementary Figure 3). In terms of the TPR, ECs performed better than transcripts, but worse than exons when using the union of all methods as the truth set. In the Bottomly analysis, Salmon was used as a representative method for transcript abundance estimation. We also performed the analysis with Kallisto ^7^, which gave results consistent with Salmon (Supplementary Figure 5 ^15^).

To evaluate the performance of ECC testing on a different simulation scheme, we ran the ECC, transcript count (using Salmon) and exon count-based (using DEXSeq-count) methods on the Love *et al.* ^13^ data. Here we found that ECC and transcript count performance was similar, with exon count analysis faring significantly worse. The simulations were based on baseline abundances derived using Salmon, which may have favoured Salmon-derived transcript quantifications in the downstream analysis. There results, taken together with the Soneson simulation and the Bottomly date, indicate that ECCs perform as well as the best method regardless of the assumptions and biases in the datasets. 

### Computational performance

While the performance of EC counts in term of sensitivity and FDR are only slightly better than exons level counts in the Soneson and Bottomly data sets, another advantage of using ECs for analysis is the speed of alignment. The process can be broken down into workflow components that include alignment of sequenced reads, quantification and testing. Table 1 shows the compute times for all three methods on the Soneson and Bottomly datasets broken down into workflow components. For the exon counting method, STAR was used for the alignment of reads to the genome (see Methods). In every case, the transcript quantification method had the fastest total run time followed by ECs and then exons. The difference was mainly driven by the speed of using pseudo alignment for transcript and EC quantification, indicating that for larger datasets the speed of analysis will be significantly faster for our proposed EC based method compared with traditional exon counting methods. A small amount of extra time was also needed for the EC method for matching EC counts to genes. In addition, DEXSeq generally runs more slowly with larger numbers of counting bins, which is the case for ECs compared with transcripts and improved scalability of DTU approaches is likely to narrow this performance gap. The speed of featureCounts over DEXseq’s counting significantly improved run times for the exon-based method; however, the total run times still lagged behind the pseudo-alignment methods. We also note that the transcript-abundance inference stage performed by pseudo-aligners is not necessary for EC-based DTU testing, making salmon slightly faster to run when quantification is skipped ( Table 1).

**Table 1.** Comparison of compute times. Compute times shown in hh:mm:ss for the simulated data (101 bp paired-end) and Bottomly (76 bp single-end) read data, with each sample aligned and quantified in serial with access to 256GB RAM and 8 cores per sample, and post-quantification steps performed on count data from all samples from each batch in a single run with 256GB RAM and 8 cores. The alignment and quantification steps show the total time taken for all samples (i.e. the serial runtime). The drosophila and human samples contained approximately 25M and 40M reads respectively, and the Bottomly samples contained approximately 16M reads. Exons counts were quantified using DEXSeq-count (ds) and featureCounts (fc).

Data	Compute times, hh:mm:ss			
drosophila	Transcripts	ECs	Exons (ds)	Exons (fc)
Alignment	-	-	03:10:34	03:10:34
Quantification	00:09:47	00:09:09	02:48:45	00:00:53
Match ECs	-	00:00:18	-	-
DEXSeq DTU	00:01:17	00:03:28	00:03:16	00:02:47
**Total**	**00:11:04**	**00:12:55**	**06:02:35**	**03:14:14**
**hsapiens**				
Alignment	-	-	01:16:33	01:16:33
Quantification	00:15:59	00:13:06	04:50:37	00:01:42
Match ECs	-	00:01:14	-	
DEXSeq DTU	00:04:54	00:27:07	00:15:53	00:30:08
**Total**	**00:20:53**	**00:41:27**	**06:23:03**	**01:48:23**
**mouse (Bottomly)**				
Alignment	-	-	00:43:12	00:43:12
Quantification	00:16:32	00:12:25	02:53:01	00:01:29
Match ECs	-	00:00:51	-	
DEXSeq DTU	00:08:49	00:25:08	00:34:53	00:44:59
Total	00:25:21	00:38:24	04:11:06	01:29:40

We also considered peak RAM usage (shown in Supplementary Table 1 ^15^), and alignment was found to use the most RAM. Overall, methods utilising pseudo alignment required significantly lower memory compared with traditional alignment. For the most RAM intensive dataset, the human simulation, exon counting required 29 GB compared to 10 GB for ECs and 5 GB for estimated transcript abundances.

## Discussion

DTU detection has previously been approached by either testing for changes to the read counts across exons or changes in the relative abundance of transcripts. These approaches are intuitive but are not necessarily optimal for short read data analysis. In particular, individual exons are not necessarily the optimal unit of isoform quantification as there are often many more exons than transcripts. In addition, transcript quantification can be difficult because read assignment is ambiguous. Fortunately, transcript quantification methods generate equivalence class counts as a forestep to estimating abundances. We propose that equivalence classes are the optimal unit for performing count based differential transcript usage testing. Equivalence class counts benefit from the advantages of both exon and transcript counts: they can be generated quickly through pseudo-alignment, there are fewer expressed than exons, and they retain the low variance between replicates seen in exon counts compared to transcripts abundances.

Here we evaluated the use of equivalence classes as the counting unit for differential transcript usage. We used two simulated datasets from drosophila and human and one biological dataset from mouse. Our results suggest that equivalence class counts provide equal or better accuracy in DTU detection compared to exon counts or estimated transcript abundances. We also found the analysis was quick to run. To allow users to run their own analyses, we provide code to convert pseudo alignments into gene level EC annotations, and a vignette with step-by-step instructions for going from fastq files to performing DTU with ECCs (see Software Availability).

The ECs used in our evaluation are defined using only the set of transcripts for which reads are compatible. Extensions to this model have been proposed that incorporate read-level information, such as fragment length, to more accurately calculate the probability of a read arising from a given transcript ^17^. Although, we do not consider probability-based equivalence classes in this work, incorporating this information for DTU deserves exploration in future work. In addition, ECCs may be calculated from full read alignment rather than pseudo-alignment ^18, 19^, which has the potential to improve accuracy further. In this work, we limited our investigation to comparing the best counting metric preceding DTU statistical testing, using DEXSeq as a representative method. Evaluation of statistical testing methods for DTU is outside the scope of this manuscript and would require further work.

One limitation to consider is transcriptome reference completeness. Pseudo-alignment is dependent on the reference, and therefore unannotated, or poorly annotated transcripts may influence downstream results. Additionally, interpretation of the results may be more difficult with equivalence classes compared with exon and transcript-based approaches. Although we can detect DTU at the gene-level, it is not simple to determine which isoforms have changed abundance without further work. We propose that superTranscripts ^20^ or Sashimi plots ^21^, which are methods for visualising the transcriptome, could be used for interpretation. Alternatively, transcript abundances, which are generated together with ECCs, can still be used to provide insight into the isoform switching.

Finally, in this work, we have focused on differential transcript usage, but ECCs have the potential to be useful in a range of other expression analysis. ECCs have already been applied to areas such as clustering and dimensionality reduction ^10^, gene-level differential expression ^12^, single-cell transcriptomics ^10, 11^ and fusion detection ^22^. We foresee that equivalence classes could serve as a base unit of measurement in many other types of analyses.

## Methods

We detected sequence content bias in the Bottomly RNA-seq data using FastQC v0.11.4, and therefore performed trimming using Trimmomatic ^23^ 0.35, using recommended parameters (2:30:10 (<seed mismatches>:<palindrome clip threshold>:<simple clip threshold>) MINLEN:36 LEADING:3 TRAILING:3 SLIDINGWINDOW:4:15). The simulated Soneson data was not trimmed.

To obtain transcript abundance counts, Salmon ^8^ v0.13.0 (development version) was run on the drosophila, human, Love and Bottomly datasets in quant mode using default parameters. Transcript-level count estimates was obtained using tximport ^24^ using the ‘scaledTPM’ scaling option. To obtain ECCs, the *--dumpEq* argument was used, as well as the *--skipQuant* to skip the quantification step. Kallisto ^7^ 0.43.0 was run in *pseudo* mode with the *--batch* argument to run all samples simultaneously. Fragment length and standard deviation were estimated from the read lengths of a single sample ( SRR099223) from the Bottomly data (len = 68, sd = 15). Equivalence classes were then matched between samples and compiled into a matrix using the python scripts (create_salmon_ec_count_matrix.py and create_kallisto_ec_count_matrix.py), available on GitHub and archived on Zenodo ^15^. Equivalence classes mapping to more than a single gene were removed. No other filtering was performed on any of the data types.

To perform the exon-based counts, raw reads were first aligned using STAR ^25^ v2.5.2a with default parameters, then the DEXSeq-count annotation was prepared excluding overlapping exon-parts, from different genes, on the same strand (--aggregate=‘no’). DEXSeq-count was then run using default parameters to obtain read counts for the counting bins specified in the GTF reference. Filtering tests on the Soneson data were performed using DRIMSeq ^26^ with min_samps_feature_expr = 3, min_feature_expr = 10, min_samps_gene_expression = 6 and min_gene_expr = 10. The same genome and transcriptome references for drosophila and human were used as in Soneson *et al.* ^6^, with only protein-coding transcripts considered for the Salmon index (as the simulations only considered protein-coding genes). For the Bottomly data, we used the NCBIM37 mm9 mouse genome and Ensembl release 67 transcriptome. Non-protein-coding transcripts were filtered out, as with the Soneson transcriptome reference in order to keep the references comparable. The samples used in the Bottomly iteration experiments were checked to ensure each sample was used in at least one iteration. We used the Gencode v28 transcriptome reference, and hg38 for the genome reference for the Love data. DEXSeq v1.26 was used to run all DTU analyses, and the *perGeneQValue* function was used to obtain gene-level FDR values from features. Cross-replicate log2(var / mean) calculations were performed on count-per-million-transformed data with light filtering (at least one sample had to have a CPM >= of 1 per feature). Compute times and RAM usage were calculated per process using /usr/bin/time -v.

An earlier version of this article can be found on bioRxiv (DOI: https://doi.org/10.1101/501106).

## Session information

The following shows the session data for the environment used to generate the paper figures:



                    ─ Session info ──────────────────────────────────────────────────────────────────────────────────────────────────────────
 setting  value                       
 version  R version 3.5.0 (2018-04-23)
 os       CentOS release 6.7 (Final)  
 system   x86_64, linux-gnu           
 ui       RStudio                     
 language (EN)                        
 collate  en_US.UTF-8                 
 ctype    en_US.UTF-8                 
 tz       Australia/Melbourne         
 date     2019-04-16                  

─ Packages ──────────────────────────────────────────────────────────────────────────────────────────────────────────────
 package              * version   date       lib source                            
 acepack                1.4.1     2016-10-29 [2] CRAN (R 3.5.0)                    
 annotate               1.58.0    2018-05-15 [2] Bioconductor                      
 AnnotationDbi        * 1.42.1    2018-05-15 [2] Bioconductor                      
 assertthat             0.2.1     2019-03-21 [1] CRAN (R 3.5.0)                    
 backports              1.1.2     2017-12-13 [2] CRAN (R 3.5.0)                    
 base64enc              0.1-3     2015-07-28 [2] CRAN (R 3.5.0)                    
 bindr                  0.1.1     2018-03-13 [2] CRAN (R 3.5.0)                    
 bindrcpp               0.2.2     2018-03-29 [2] CRAN (R 3.5.0)                    
 Biobase              * 2.40.0    2018-05-15 [2] Bioconductor                      
 BiocGenerics         * 0.26.0    2018-05-15 [2] Bioconductor                      
 BiocParallel         * 1.14.2    2018-07-08 [2] Bioconductor                      
 biomaRt                2.36.1    2018-06-05 [2] Bioconductor                      
 Biostrings             2.48.0    2018-05-15 [2] Bioconductor                      
 bit                    1.1-14    2018-05-29 [2] CRAN (R 3.5.0)                    
 bit64                  0.9-7     2017-05-08 [2] CRAN (R 3.5.0)                    
 bitops                 1.0-6     2013-08-17 [2] CRAN (R 3.5.0)                    
 blob                   1.1.1     2018-03-25 [2] CRAN (R 3.5.0)                    
 checkmate              1.8.5     2017-10-24 [2] CRAN (R 3.5.0)                    
 cli                    1.1.0     2019-03-19 [1] CRAN (R 3.5.0)                    
 cluster                2.0.7-1   2018-04-13 [2] CRAN (R 3.5.0)                    
 colorspace             1.3-2     2016-12-14 [2] CRAN (R 3.5.0)                    
 crayon                 1.3.4     2017-09-16 [2] CRAN (R 3.5.0)                    
 data.table           * 1.11.4    2018-05-27 [2] CRAN (R 3.5.0)                    
 DBI                    1.0.0     2018-05-02 [2] CRAN (R 3.5.0)                    
 DelayedArray         * 0.6.5     2018-08-15 [2] Bioconductor                      
 DESeq2               * 1.20.0    2018-06-18 [2] Bioconductor                      
 DEXSeq               * 1.26.0    2018-07-19 [1] Bioconductor                      
 digest                 0.6.16    2018-08-22 [2] CRAN (R 3.5.0)                    
 dplyr                * 0.7.6     2018-06-29 [2] CRAN (R 3.5.0)                    
 DRIMSeq              * 1.8.0     2018-10-30 [1] Bioconductor                      
 edgeR                * 3.22.3    2018-06-21 [2] Bioconductor                      
 evaluate               0.11      2018-07-17 [2] CRAN (R 3.5.0)                    
 foreign                0.8-71    2018-07-20 [2] CRAN (R 3.5.0)                    
 formatR                1.5       2017-04-25 [2] CRAN (R 3.5.0)                    
 Formula                1.2-3     2018-05-03 [2] CRAN (R 3.5.0)                    
 futile.logger        * 1.4.3     2016-07-10 [2] CRAN (R 3.5.0)                    
 futile.options         1.0.1     2018-04-20 [2] CRAN (R 3.5.0)                    
 genefilter             1.62.0    2018-06-18 [2] Bioconductor                      
 geneplotter            1.58.0    2018-05-15 [2] Bioconductor                      
 GenomeInfoDb         * 1.16.0    2018-05-15 [2] Bioconductor                      
 GenomeInfoDbData       1.1.0     2018-05-15 [2] Bioconductor                      
 GenomicRanges        * 1.32.6    2018-07-20 [2] Bioconductor                      
 ggplot2              * 3.0.0     2018-07-03 [2] CRAN (R 3.5.0)                    
 glue                   1.3.0     2018-07-17 [2] CRAN (R 3.5.0)                    
 gridExtra            * 2.3       2017-09-09 [2] CRAN (R 3.5.0)                    
 gtable                 0.2.0     2016-02-26 [2] CRAN (R 3.5.0)                    
 Hmisc                  4.1-1     2018-01-03 [2] CRAN (R 3.5.0)                    
 hms                    0.4.2     2018-03-10 [2] CRAN (R 3.5.0)                    
 htmlTable              1.12      2018-05-26 [2] CRAN (R 3.5.0)                    
 htmltools              0.3.6     2017-04-28 [2] CRAN (R 3.5.0)                    
 htmlwidgets            1.2       2018-04-19 [2] CRAN (R 3.5.0)                    
 httr                   1.4.0     2018-12-11 [1] CRAN (R 3.5.0)                    
 hwriter                1.3.2     2014-09-10 [2] CRAN (R 3.5.0)                    
 IRanges              * 2.14.11   2018-08-24 [2] Bioconductor                      
 knitr                  1.20      2018-02-20 [2] CRAN (R 3.5.0)                    
 lambda.r               1.2.3     2018-05-17 [2] CRAN (R 3.5.0)                    
 lattice                0.20-35   2017-03-25 [2] CRAN (R 3.5.0)                    
 latticeExtra           0.6-28    2016-02-09 [2] CRAN (R 3.5.0)                    
 lazyeval               0.2.1     2017-10-29 [2] CRAN (R 3.5.0)                    
 limma                * 3.36.2    2018-06-21 [2] Bioconductor                      
 locfit                 1.5-9.1   2013-04-20 [2] CRAN (R 3.5.0)                    
 magrittr               1.5       2014-11-22 [2] CRAN (R 3.5.0)                    
 Matrix                 1.2-14    2018-04-09 [2] CRAN (R 3.5.0)                    
 matrixStats          * 0.54.0    2018-07-23 [2] CRAN (R 3.5.0)                    
 memoise                1.1.0     2017-04-21 [2] CRAN (R 3.5.0)                    
 munsell                0.5.0     2018-06-12 [2] CRAN (R 3.5.0)                    
 nnet                   7.3-12    2016-02-02 [2] CRAN (R 3.5.0)                    
 pillar                 1.3.0     2018-07-14 [2] CRAN (R 3.5.0)                    
 pkgconfig              2.0.2     2018-08-16 [2] CRAN (R 3.5.0)                    
 plyr                   1.8.4     2016-06-08 [2] CRAN (R 3.5.0)                    
 prettyunits            1.0.2     2015-07-13 [2] CRAN (R 3.5.0)                    
 progress               1.2.0     2018-06-14 [2] CRAN (R 3.5.0)                    
 purrr                  0.2.5     2018-05-29 [2] CRAN (R 3.5.0)                    
 R6                     2.4.0     2019-02-14 [1] CRAN (R 3.5.0)                    
 RColorBrewer         * 1.1-2     2014-12-07 [2] CRAN (R 3.5.0)                    
 Rcpp                   0.12.18   2018-07-23 [2] CRAN (R 3.5.0)                    
 RCurl                  1.95-4.11 2018-07-15 [2] CRAN (R 3.5.0)                    
 readr                * 1.1.1     2017-05-16 [2] CRAN (R 3.5.0)                    
 reshape2               1.4.3     2017-12-11 [2] CRAN (R 3.5.0)                    
 rlang                  0.2.2     2018-08-16 [2] CRAN (R 3.5.0)                    
 rmarkdown              1.10.2    2018-06-18 [2] Github (rstudio/rmarkdown@18207b9)
 rpart                  4.1-13    2018-02-23 [2] CRAN (R 3.5.0)                    
 rprojroot              1.3-2     2018-01-03 [2] CRAN (R 3.5.0)                    
 Rsamtools              1.32.3    2018-08-22 [2] Bioconductor                      
 RSQLite                2.1.1     2018-05-06 [2] CRAN (R 3.5.0)                    
 rstudioapi             0.10      2019-03-19 [1] CRAN (R 3.5.0)                    
 S4Vectors            * 0.18.3    2018-06-18 [2] Bioconductor                      
 scales                 1.0.0     2018-08-09 [2] CRAN (R 3.5.0)                    
 sessioninfo            1.1.1     2018-11-05 [1] CRAN (R 3.5.0)                    
 statmod                1.4.30    2017-06-18 [2] CRAN (R 3.5.0)                    
 stringi                1.2.4     2018-07-20 [2] CRAN (R 3.5.0)                    
 stringr                1.3.1     2018-05-10 [2] CRAN (R 3.5.0)                    
 SummarizedExperiment * 1.10.1    2018-05-15 [2] Bioconductor                      
 survival               2.42-6    2018-07-13 [2] CRAN (R 3.5.0)                    
 tibble                 1.4.2     2018-01-22 [2] CRAN (R 3.5.0)                    
 tidyselect             0.2.4     2018-02-26 [2] CRAN (R 3.5.0)                    
 tximport             * 1.8.0     2018-05-15 [2] Bioconductor                      
 VennDiagram          * 1.6.20    2018-03-28 [2] CRAN (R 3.5.0)                    
 withr                  2.1.2     2018-03-15 [2] CRAN (R 3.5.0)                    
 XML                    3.98-1.16 2018-08-19 [2] CRAN (R 3.5.0)                    
 xtable                 1.8-3     2018-08-29 [2] CRAN (R 3.5.0)                    
 XVector                0.20.0    2018-05-15 [2] Bioconductor                      
 yaml                   2.2.0     2018-07-25 [2] CRAN (R 3.5.0)                    
 zlibbioc               1.26.0    2018-05-15 [2] Bioconductor                      

[1] /home/marek.cmero/R/x86_64-pc-linux-gnu-library/3.5
[2] /usr/local/installed/R/3.5.0/lib64/R/library
                


## Data availability

### Underlying data

The Soneson *et al.* ^6^ drosophila and human simulation data was obtained from ArrayExpress repository, accession number E-MTAB-3766.

Truth data was obtained from http://imlspenticton.uzh.ch/robinson_lab/splicing_comparison/, files diff_splicing_comparison_drosophila.zip and diff_splicing_comparison_human.zip.

The Bottomly *et al.* ^14^ dataset was obtained from the NCBI Sequence Read Archive, accession number SRP004777.

The Love *et al.* dataset was obtained from:

https://doi.org/10.5281/zenodo.1291375 ^27^

https://doi.org/10.5281/zenodo.1291404 ^28^

https://doi.org/10.5281/zenodo.1291443 ^29^

The Love *et al*. ^15^ data feature counts are available from:

https://doi.org/10.5281/zenodo.2644723 ^30^

All other feature count data is available in the ec-dtu-paper repository ^31^.

### Extended data

Zenodo: Supplementary Material for "Fast and accurate differential transcript usage by testing equivalence class counts". https://doi.org/10.5281/zenodo.2644649 ^15^. The following extended data are available:

- Supplementary Figure 1: Shows the dispersion versus mean normalised counts for all features across the three data sets, generated using DEXSeq’s ‘plotDispEsts’ function. As described in Love *et al*., the red line shows the fitted dispersion-mean trend, the blue dots indicate the shrunken dispersion estimates, and the blue circles indicate outliers not shrunk towards the prior.
- Supplementary Figure 2: Shows the significant genes (FDR < 0.05) shared between the methods, obtained from DEXSeq run on the full Bottomly *et al*. data set for each feature.
- Supplementary Figure 3: Shows the ability of the three methods to recreate the results of a full comparison (10 vs. 11) of the Bottomly *et al*. data using random subsets of 3 vs. 3 samples across 20 iterations using FDR < 0.05 (FDR = 0.05 is indicated by the dotted line). The lines between the plots join data points from the same iteration. Each row uses a different ‘truth’ set: union is the set of genes called significant by any method, intersect is the set of genes called significant by all methods, and individual is the set of genes called significant by that method only.
- Supplementary Figure 4: The number of false positives versus each gene’s rank (by FDR) for one iteration (3 vs. 3) of the Bottomly subset tests for the top 500 genes. The union of significant genes across all methods was used as the truth set.
- Supplementary Figure 5: Kallisto versus Salmon’s performance on the Bottomly subset testing experiments, using each method’s significant genes from the full (10 vs. 11) run as the truth set for calculating both metrics.
- Supplementary Figure 6: Performance of ECC, transcript count (using Salmon) and exon count (using DEXSeq-count) methods on the Soneson data with and without DRIMSeq filtering (see paper methods for full filtering criteria).
- Supplementary Figure 7: An example EC usage plot for a single gene from the hsapiens DECU results. Log EC usage is shown across each equivalence class for both conditions. Yellow blocks indicate significant ECs. As ECs do not correspond easily to genomic locations, no special ordering is applied the the ECs. In the given example, transcripts mapping to each significant EC can be observed in the data. The significant ec142580 corresponds to a single transcript (ENST00000443443), indicating that interpretation can be straight-forward if the EC is associated with a single transcript. See Section 6.4 of the EC DTU vignette for how to run the plotting code.
- Supplementary Figure 8: Performance of ECC, transcript count (using Salmon) and exon count (using DEXSeq-count) methods on the Love *et al*. data (12 vs. 12 samples). The points show nominal FDR cutoffs of 0.01, 0.05 and 0.1.
- Supplementary Table 1: Maximum RAM usage for each job in GB. Each task was run as specified in the compute times table in the main paper ( Table 1).
- Supplementary Table 2: Average number of exons and transcripts per gene from the hg38 ensembl reference.

Extended data are available under the terms of the Creative Commons Attribution 4.0 International license (CC-BY 4.0).

## Software availability

**Pipeline used to reproduce the quantification data generated in this paper:** https://github.com/Oshlack/ec-dtu-pipe.

**Archived source code at time of publication:** https://doi.org/10.5281/zenodo.2644725 ^31^.

**Source code to run the analyses and generate the paper figures:** https://github.com/Oshlack/ec-dtu-paper.

Vignette for running DTU analyses using ECCs:

https://github.com/Oshlack/ec-dtu-paper/wiki/Vignette

**Archived source code at time of publication:** https://doi.org/10.5281/zenodo.2644724 ^32^.

**License:** MIT license.
