# Supplementary material for: Using equivalence class counts for fast and accurate testing of differential transcript usage
Source: F1000Res. 2019 Apr 29;8:265. Originally published 2019 Mar 7. [Version 2] doi: 10.12688/f1000research.18276.2 (PMC6524746; doi:10.12688/f1000research.18276.2)
Supplement: Peer review history

# Peer review: Reviewer response for version 2

**DOI:** 10.5256/f1000research.20869.r47844

**Published:** 2019-05-28

**Authors:** Alejandro Reyes

**Affiliations:** 1 Department of Biostatistics and Computational Biology, Dana-Farber Cancer Institute, Boston, MA, USA

In the revised version of the manuscript, the authors have addressed all the concerns I raised in the previous version. The conclusions are well-supported by data and scientifically sound.

I have read this submission. I believe that I have an appropriate level of expertise to confirm that it is of an acceptable scientific standard.

# Peer review: Reviewer response for version 2

**DOI:** 10.5256/f1000research.20869.r47843

**Published:** 2019-05-15

**Authors:** Leonardo Collado-Torres

**Affiliations:** 1 Lieber Institute for Brain Development, Baltimore, MD, USA

In version 2 of their paper, the authors thoroughly and comprehensively addressed my minor points from version 1. I appreciate the effort the authors did to improve the reproducibility of their work thanks to the R session information and their vignette provided in response to another reviewer ^1^. Given this, I'm happy to approve their article for indexing.

 My only minor comment is that it would have been ideal to mention in the amendments from Version 1 that featureCounts and kallisto were removed from Figure 3. This change initially confused me, given my version 1 comment on the color issue between `featurecounts_flat` and `salmon`. I have checked that this change in Figure 3 does not affect the rest of the main text.

 Anyhow, for other readers: this change in Figure 3 was requested by one of the reviewers and the authors complied in their response ^2^.

I have read this submission. I believe that I have an appropriate level of expertise to confirm that it is of an acceptable scientific standard.

## References

[1] ReyesA: Referee Report For: Fast and accurate differential transcript usage by testing equivalence class counts [version 1; peer review: 3 approved with reservations]. *F1000Research*.8(265) : https://doi.org/10.5256/f1000research.19992.r45466 10.5256/f1000research.19992.r45466

[2] Vitting-SeerupK: Referee Report For: Fast and accurate differential transcript usage by testing equivalence class counts [version 1; peer review: 3 approved with reservations]. *F1000Research*.8(265) : https://doi.org/10.5256/f1000research.19992.r45465 10.5256/f1000research.19992.r45465

# Peer review: Reviewer response for version 2

**DOI:** 10.5256/f1000research.20869.r47845

**Published:** 2019-05-14

**Authors:** Kristoffer Vitting-Seerup

**Affiliations:** 1 Department of Biology, University of Copenhagen, Copenhagen, Denmark

**Summary**

 In this manuscript Cmero *et al.* compares a TCC based DTU workflow against at transcript based and an exon based workflow using both simulated and real data reaching the conclusion that a TCC based workflow is superior – a novel and important finding. The manuscript is overall well presented and the approach is state-of-the art. Unfortunately the actual analysis suffers from major technical problems which undermines the findings presented. Importantly these problems were NOT fixed in the second edition!

**Major comments**:

1. Except for Figure S6 all analysis presented is still performed on unfiltered data which makes the analysis untrustworthy. Therefore please note:
  - The lack of expression filtering will affect all analysis presented since many lowly or zero expressed features will be included thereby skewing the global comparison due to the difference in the proportion of low/zero in the different datasets compared (as also pointed out in reference 1 ^1^). 
  - This approach does reflect a typical RNA-seq analysis workflows which always include a step which filters out lowly expressed features before continued analysis.
  - Therefore, the authors should include (or even better replace) all the current analysis with an analysis based on dataset which have been pre-filtered for expression.
  - For inspiration of expression filtering see ^1^^,^^2^, the edgeR::filterByExpr() function or use the classical 1 TPM cutoff.
  - Naturally the 3 dataset should be also filtered to be comparable (same transcripts/genes tested with all methods).
2. If, as indicated in the methods, the authors actually used the read length identified directly from the Bottomly *et al.* dataset as the fragment length in the Salmon quantification the entire analysis of the Bottomly *et al.* dataset is not trustworthy since read-length != fragment-length (!). Using wrong fragment length will disproportionally affect the the Salmon based DTU analysis (not the exon and less the ECC based approaches since they do not rely on the TPM estimation affected by the fragment length). The authors really need to elaborate on this and justify the quantification approach!
3. I really appreciate that the Love *et al.* 2018 data is now also included – but it seems strange strangely selective that it is not included in the main figures and the there is no analysis similar to Fig 2. Furthermore, the authors should (also) include an analysis of Love *et al.* data using only 3 replicates – else the results are not comparable to the Bottomly and Sonneson results.

**Minor comments**:

 Generally:

- The authors should use “transcript compatibility counts” (TCC) (aka not “equivalence class read counts” (EC) and derivations thereof) since TCC is the terminology used in the field when ECs are used for quantification ^3^.

Introduction

- In addition to exon and transcript based analysis approaches the authors also need to mention analysis of individual splice events (via tools such as SUPPA2 and rMATS) as well as the types of analysis which groups multiple features together (such as Leafcutter and MAJIQ) to make clear that there are 4 different approaches (with TCC based approach being a fifth (or a deviation of transcript based)). I do not require the authors to also compared the TCC based approach to the two omitted workflows – but they should be mentioned in the introduction for completeness.
- The authors should cite ^3^ for the term “transcript compatibility count”.

Results

- Please also show the average TPR vs FDR plot (similar to Fig 3a) for the Bottomly *et al.* data as interpreting these measures together is necessary for a fair comparison.
- The authors should discuss the much larger variance in FDR for TCC and exon based approaches.

Methods

- Please provide the unfiltered salmon quantification results (the “quant.sf” files) from the Bottomly *et al.* data as supplementary files to facilitate reproducibility.

I have read this submission. I believe that I have an appropriate level of expertise to confirm that it is of an acceptable scientific standard, however I have significant reservations, as outlined above.

## References

[1] YiLLiuLMelstedPPachterL: A direct comparison of genome alignment and transcriptome pseudoalignment. *bioRxiv*.2018; 10.1101/444620 10.1101/444620

[2] LoveMISonesonCPatroR: Swimming downstream: statistical analysis of differential transcript usage following Salmon quantification. *F1000Res*.2018;7: 10.12688/f1000research.15398.3 952 10.12688/f1000research.15398.3 30356428PMC6178912

[3] NtranosVKamathGZhangJPachterLTseD: Fast and accurate single-cell RNA-seq analysis by clustering of transcript-compatibility counts. *Genome Biology*.2016;17(1) : 10.1186/s13059-016-0970-8 10.1186/s13059-016-0970-8 27230763PMC4881296

# Peer review: Reviewer response for version 1

**DOI:** 10.5256/f1000research.19992.r45467

**Published:** 2019-04-12

**Authors:** Leonardo Collado-Torres

**Affiliations:** 1 Lieber Institute for Brain Development, Baltimore, MD, USA

In this manuscript the authors Marek Cmero, Nadia M. Davidson and Alicia Oshlack describe in detail their proposed approach for identifying genes with differential transcript usage (DTU, particularly isoform switching) using equivalence classes obtained through pseudo-alignment methods such as Salmon and Kallisto. By doing so, the authors leverage the computational advantages of pseudo-alignment methods, particularly speed and RAM requirements, together with statistical methods initially developed for differential exon usage (mainly DEXSeq) to identify genes with DTU events at a comparable (or even lower) error rates than exon based analyses which are more precise than transcript-level analyses. That is, their proposed method is fast, has low computational requirements (measured by RAM usage), and has error rates comparable if not better than state of the art alternatives. If time and computational resources are not limiting factors, the method the authors propose still gains an advantage over exon based methods by taking advantage of the nature of the human and mouse transcriptomes where genes can have more exons than transcripts, thus leading to power gains by their method. However, as presented their method also relies on a correct annotation of the transcriptome since un-annotated isoforms that involve new exons or new exon boundaries could potentially affect the results.

 Nevertheless, I think that it should be possible to apply their method in combination with others in order to minimize this issue. Overall the authors of this manuscript did an excellent job explaining their new method, comparing against earlier work, and explaining the different implications of their work. I look forward to their future software for applying this method as https://github.com/Oshlack/ec-dtu-paper has all the foundations for making an R/Bioconductor package.

**Minor points**

- Figures 2 and 3 are missing labels for each sub-panel. For example, the legend for Figure 2 talks about (a) and (b) and while one can assume that the top panel is (a), it's best to be explicit about this type of information.
- Figure 2 top panel. Maybe show the data points in case the boxplots mask some information about the distribution. See here for some code by Rafael Irizarry or here for longer code examples that I wrote. If it looks like a bell-shaped distribution, then I think that it could be okay to simply mention that in the text (in the case that the figure has many points and you prefer not to include it). From Figure 3 bottom panel, I can see that you already plotted the points in that case.
- Figure 2 bottom panel. This also has some code for showing density plots with little bars in the bottom for the observed points.
- Page 5, bottom left. "Count variability of ECs was on average closer to the exon count variability distribution than ECs." is incorrect. I believe that it should read "Count variability of ECs was on average closer to the exon count variability distribution than transcripts".
- Figure 3, top panel. I can't distinguish the colors between `featurecounts_flat` and `salmon`. 
- Figure 3, top panel. I don't know what the dotted lines represent: maybe FDR 0.01, 0.05 and 0.1?
- Figure 3, top panel. You might want to consider annotating with text the highest TPR point for each dataset which is quoted in the text in page 5 right side.
- I appreciate that Figure 3 (top panel) shows the full range, but maybe it would be useful to have a zoomed-in version in the supplementary material in order to see the differences more clearly. Maybe have a ylim from 0.5 to 0.8, and an xlim from 0 to 0.6 (or something like that).
- Page 6, bottom left. "ECs called the highest number of genes with significant DTU (1485 genes, in contrast to the 748 and 391 genes called significant by the transcript and exon-based methods respectively)." That sentence is incorrect based on Supplementary Figure 2. The numbers for genes with significant DTU match for the transcript and exon based methods, but they don't for the EC based method since 228 + 204 + 147 + 96 = 675. This numerical change affects the conclusions drawn from Supplementary Figure 2. 
- Page 6, bottom right. Were all samples from the full bottomly dataset used in any of the 20 iterations? Or were there some samples that were used in many of the replications? With 20 iterations I guess that there's a small chance that some samples were under-represented or over-represented in the iterations.
- Figure 3, bottom panel. I really liked the lines you show in Supplementary Figure 3 to identify the different comparable replicates. The lines helped me visualize that the ranks were consistent across replicates since the lines rarely intersect each other. I suggest mentioning those lines in the caption for Figure 3 where you refer to Supplementary Figure 3, or maybe even swapping the panels from Figure 3 (bottom) for the equivalent ones from Supplementary Figure 3 (no need to change Figure S3 in that case, that is, it's okay to repeat the panels).
- Page 7, right side. Typo "psuedo-" instead of "pseudo-".
- Page 8, left side. "We also found the analysis was quick to run and we provide code to convert [...]". I highly recommend including the URL here for the code or mention in a parenthesis in which section of the paper can one find the link to the code.
- Page 8, right side. I recommend also citing the Bottomly et al paper when you mention that the data was downloaded from SRR099223. You already cite the paper in other parts of your manuscript.
- From the link to the bioRxiv pre-print I was able to find tweets citing the pre-print and have to agree with this tweet saying "this type of stuff is what the field needs".
- Here, I didn't find the actual versions of the packages used. I suggest including the output of "options(width = 120); sessioninfo::session_info()" somewhere in that repository.
- I think that you don't need to call gc() manually in your function calls here. Normally R takes care of it.
- Since I see here that 8 cores were used for your method, I'm curious now looking at Supplementary Table 1 if the RAM presented there is by thread (core) or by process, and if so, how many cores were used for the other steps.

I have read this submission. I believe that I have an appropriate level of expertise to confirm that it is of an acceptable scientific standard, however I have significant reservations, as outlined above.

## References

[1] PatroRDuggalGLoveMIIrizarryRAKingsfordC: Salmon provides fast and bias-aware quantification of transcript expression. *Nat Methods*.2017;14(4) : 10.1038/nmeth.4197 417-419 10.1038/nmeth.4197 28263959PMC5600148

[2] BrayNLPimentelHMelstedPPachterL: Near-optimal probabilistic RNA-seq quantification. *Nat Biotechnol*.34(5) : 10.1038/nbt.3519 525-7 10.1038/nbt.3519 27043002

[3] AndersSReyesAHuberW: Detecting differential usage of exons from RNA-seq data. *Genome Res*.2012;22(10) : 10.1101/gr.133744.111 2008-17 10.1101/gr.133744.111 22722343PMC3460195

# Response

**Published:** 2019-04-19

**Authors:** Alicia Oshlack

**Affiliations:** Murdoch Childrens Research Institute, Australia

Thank you for taking the time to review our paper and for the helpful suggestions.

Minor points:

- We have fixed the issues with Figures 2 and 3, and have added a description of the dotted lines in Figure 3a.
- We now use more distinguishable colour-palettes in all cases where colours identify data points.
- Supplementary Figure 6 has been added (showing filtered vs. unfiltered results), and uses zoomed-in axes. This plot contains the original results for ECC, exon and transcript counts, which should now be easier to distinguish.
- We agree with the reviewer that boxplots can mask information. However, due to the discrete nature of the data, combined with the log-scale, results in a stepwise artefact. Please see Soneson *et al.*[1] Supplementary Figure 3 for an example. We have therefore opted to retain boxplots. We note that the source code for generating these plots is available in the ec-dtu-paper github repository should readers want to inspect the raw data.
- We also like the suggestion of the density ridges, however, due to the high number of data points (>1 million), this did not add any additional information to the visualisation.
- Instead of annotating TPR points for clarity on the plots in Figure 3a, we have added Supplementary Figure 6, which contains the same data points for ECs, transcripts and exons, as well as their respective performance using filtered features.
- We inspected the random samples selected for the Bottomly analyses (we have provided random seeds in the R markdown notebook) and noted that all samples were used at least one time. We have added code to the paper R markdown notebook to show sample usage across iterations. As is apparent in Supplementary Figure 3, the usage of particular samples is less important relative to the performance ranks observed of the method types across the iterations.
- The number of significant genes found, reflected in Supplementary Figure 2, has been corrected in the main text.
- We now mention the lines between replications in Figure 3’s caption.
- We have added all suggested links, references and fixed the typos pointed out in the paper.
- Session info has been added to the main paper, and we have removed the gc() statements from the code.
- Supplementary Table 1 lists RAM by process; this has been clarified in the caption.

 

References:

[1] Soneson, C., Matthes, K. L., Nowicka, M., Law, C. W., & Robinson, M. D. (2016). Isoform prefiltering improves performance of count-based methods for analysis of differential transcript usage. *Genome Biology*, *17*(1), 1–15. https://doi.org/10.1186/s13059-015-0862-3

# Peer review: Reviewer response for version 1

**DOI:** 10.5256/f1000research.19992.r45466

**Published:** 2019-03-25

**Authors:** Alejandro Reyes

**Affiliations:** 1 Department of Biostatistics and Computational Biology, Dana-Farber Cancer Institute, Boston, MA, USA

Cmero, Davidson and Oshlack propose a novel approach to use RNA-seq data to test for differences in transcript usage between conditions. Instead of using exon-level or transcript-level counts, the authors propose using equivalence class counts (ECCs) resulting from pseudo-aligning/quasi-mapping to reference transcriptomes as input to existing methods to test for differences in exon usage. Using both simulated and real datasets, the authors show that using ECCs is comparable to using exon-level counts in terms of false discovery rates and true positive rates. They show that the ECC approach is computationally more efficient, although its results are more difficult to interpret. The analyses are reproducible and available through Github.

 The manuscript is well written and easy to follow. The whole idea is straightforward and very clever.

 Below are two suggestions for improving the implementation of the software:

1. Although some python scripts are available, they need better documentation and examples with toy datasets. From the code in the Github repository, it is not clear what steps one should follow to use the ECC approach for DTU. I would suggest writing a Bioconductor-like vignette that explains how to run kallisto/salmon with the parameters to get equivalence classes, how to use the python scripts to generate the equivalence class matrices, and how to transform these matrices into objects from the DEXSeq, DRIMseq and similar packages.
2. As the authors acknowledge, a strong limitation of the ECC approach is result interpretation, which could be improved by visualizing the ECC equivalence classes. The interpretation of the ECC approach would be much easier if the authors provide code to plot transcripts and ECC classes of a gene (as it is done in the cartoon of Figure 1) linked with the counts of each equivalence class for each sample.

Minor points:

1. It would be helpful for the reader if the authors improved figure labels and figure legends. For example, in Figure 3a, rather than just referring to the paper by Soneson et al. ^1^, I would suggest to describe what each point represents, what each axis is and how the metrics shown were defined.
2. In the introduction, the authors say “Typically, there will be more counting bins than transcripts, resulting in lower power to detect differences between samples.” Could the authors either explain further this statement or cite a reference that explains it?
3. I understand the logic behind defining a “truth set” of genes with DTU in the analysis of the real data. However, the real number of true positives is likely larger and thus the resulting metric is not strictly a true positive rate. Perhaps it would be more accurate to call it differently (see for example, Norton et al. ^2^).

I have read this submission. I believe that I have an appropriate level of expertise to confirm that it is of an acceptable scientific standard, however I have significant reservations, as outlined above.

## References

[1] SonesonCMatthesKLNowickaMLawCWRobinsonMD: Isoform prefiltering improves performance of count-based methods for analysis of differential transcript usage. *Genome Biol*.2016;17: 10.1186/s13059-015-0862-3 12 10.1186/s13059-015-0862-3 26813113PMC4729156

[2] NortonSVaquero-GarciaJLahensNGrantGBarashY: Outlier detection for improved differential splicing quantification from RNA-Seq experiments with replicates. *Bioinformatics*.2018;34(9) : 10.1093/bioinformatics/btx790 1488-1497 10.1093/bioinformatics/btx790 PMC645442529236961

# Response

**Published:** 2019-04-19

**Authors:** Alicia Oshlack

**Affiliations:** Murdoch Childrens Research Institute, Australia

Thank you for taking the time to review our paper and for the helpful suggestions.

Major comments:

- We have created a step-by-step Bioconductor-style vignette to allow users to easily reproduce ECC-based DTU testing with a toy data set. We include instructions for running each step manually, as well as an automated analysis using the ec-dtu-pipe pipeline we have developed. The vignette can be found here, which we note in the paper.
- Figure 1 in the original paper shows a highly simplified version of how ECs can be derived from a small set of transcripts and exons. In reality, genes have on average many more transcripts, exons and, consequently, equivalence classes. Furthermore, ECs may be disjoint (not connected by intervening sequence) or require a junction. As ECs are determined by kmers, creating a direct mapping between ECs and the genome is challenging. Given the complexity of ECs, even a clean mapping between EC and genome position may be difficult to interpret. Given these limitations, we have instead opted to include a simple visualisation option, similar to DEXSeq, plotting EC names and their relative log counts across conditions, per gene. Such a visualisation example can be found in Supplementary Figure 7 (note the large number of ECs present in this gene). The function to create these plots (plot_ec_usage) is found in the ec-dtu-paper repository (and is referenced in the vignette) will also print all significant ECs of the gene, and their associated transcripts. In the example, one of the significant ECs has a single associated transcript, making DTU inference relatively straight-forward.

Minor comments:

- We have added explanatory text in Figure 3a to explain the FDR/TPR plots and their respective FDR cutoffs. We have also added (a) and (b) labels for Figures 1-3.
- We have further explained the sentence about how the number of counting bins affects power. We have also added Supplementary Table 2 to illustrate this point, which shows the average number of exons and transcripts per gene for the Ensembl human gene reference.
- We have rename the TPR to ‘Fraction recalled’ (also in Supplementary Figure 3) to indicate that the metric does not strictly measure false positive rate.

# Peer review: Reviewer response for version 1

**DOI:** 10.5256/f1000research.19992.r45465

**Published:** 2019-03-20

**Authors:** Kristoffer Vitting-Seerup

**Affiliations:** 1 Department of Biology, University of Copenhagen, Copenhagen, Denmark

**Summary**

 In the manuscript “Fast and accurate differential transcript usage by testing equivalence class counts” by Cmero *et al* suggest to use the ability of modern lightweight RNA-seq aligners to produce transcript compatibility counts (TCC) in combination with standard tools designed for differential transcript usage (DTU). Although the idea, as described in the introduction of the article, have been partly touched on by previous publications from the Pachter Lab, the approach used in this manuscript is novel since it describes a direct DTU analysis whereas the previous publications only inferred DTU indirectly. In this manuscript Cmero *et al* compares a TCC based DTU workflow against at transcript based and an exon based workflow using both simulated and real data reaching the conclusion that a TCC based workflow is superior – a novel and important finding. The manuscript is overall well presented and the analysis approach is state-of-the art. Unfortunately the analysis is not quite extensive enough and it suffers from a few major technical problems which together with a general lack of clarity in the writing means the manuscript requires major revisions.

**Major comments**:

- The authors should also evaluate on the simulated data from Love *et al* 2018 ^1^ to test the effect of:
  - A different simulation scheme (since the FDRs are so high for Soneson *et al* data).
  - Investigate the stability of the results using different number of replicates
- All analysis presented is performed on unfiltered data which is problematic. Firstly it does not reflect typical RNA-seq analysis workflows which always include a step which filters out lowly expressed features before continued analysis. Furthermore, and more problematically, the lack of expression filtering will affect all analysis presented since many lowly or zero expressed features will be analyzed thereby skewing the global comparison due to the difference in the proportion of low/zero in the different datasets/pipelines ^2^. Therefore, the authors should include (or replace the current analysis with) an analysis based on dataset which have been pre-filtered for expression. For inspiration of expression filtering ^1^^,^^2^, the edgeR::filterByExpr() function or use the classical 1 TPM cutoff. Naturally the 3 dataset should be also filtered to be comparable (same transcripts/genes tested with all methods).
- To ensure correct quantification and to make the genome based (STAR) and lightweight based (Salmon) analysis comparable the Salmon index should be build from all transcripts and subsequently (after quantification) the data should be reduced to only protein coding genes. This is necessary to ensure that reads mapping to both protein coding genes and lncRNAs are correctly quantified (and are quantified in a manner comparable to the genome based approach).
- The manuscript is in general not concise enough. Throughout, the manuscript is very hard to follow which workflow is referred to and the order in which workflows they are presented is not logical (e.g. starting a section with explain about the alternative workflow does not make sense). Figures contain data never mention or used. Especially the discussion falls short of the mark as major parts are either repetitive non-informative.

**Minor comments**:

- Generally:
  - The authors should use “transcript compatibility counts” (TCC) (aka not “equivalence class read counts” (EC) and derivations thereof) since TCC is the terminology used in the field when ECs are used for quantification ^3^.
- Title:
  - The title seems to lack a word after such as “analysis” or “testing” after “differential transcript usage”
- Abstract:
  - In the sentence “However, recent evaluations show lower sensitivity in DTU analysis” I guess the authors mean compared to exon-level analysis but this needs to be specified.
  - The conclusion is to broad. The authors investigate DTU but conclude about “many” analysis. Such a sentence should probably be saved for a review paper.
- Introduction:
  - In addition to exon and transcript based analysis approaches the authors also need to mention analysis of individual splice events (via tools such as SUPPA2 and rMATS) as well as the types of analysis which groups multiple features together (such as Leafcutter and MAJIQ) to make clear that there are 4 different approaches (with TCC based approach being a fifth (or a deviation of transcript based)). I do not require the authors to also compared the TCC based approach to the two omitted workflows – but they should be mentioned in the introduction for completeness.
  - I think it could be beneficial to refer more the lower part of Figure 1 in the Introduction since it very clearly present the 3 different workflows in question?
  - The drawbacks of pseudo/quasi alignment should be mentioned/discussed either in the introduction or discussion.
  - In the sentence “Depending on its sequence, a read can align to all three transcripts, only two of the transcripts or just one transcript. These different combinations result in four possible equivalence classes, containing read counts, for this gene” the last statement is wrong. There are 6 possible (the authors omit uniquely t2 and uniquely t3). This should either be mention or it should be highlighted the example reads in Figure 1 give rise to 4 possibilities.
  - The authors should provide a reference ^3^ for the term “transcript compatibility count”.
  - The authors should also discuss the ideas presented in Ntranos *et al* 2019 ^4^ in the discussion of the Yi et al 2018 paper. Specifically the “catch-it-all” and “any transcript-level phenomena” part of the sentence in Cmero *et al*: “Yi and colleagues have recently introduced direct differential testing on equivalence classes in a catch-all method to identify genes that display any transcript-level phenomena” needs to be changed as aggregation of DTE p-values cannot identify isoform switches if the gene expression is also changing (as discussed in detail in Ntranos *et al* 2019) – hence the need for methods specifically designed for DTU detection and thereby also the need for the workflow presented by the authors Cmero *et al.*
  - For Figure 1: Could it be beneficial to divide Figure 1 into A and B referring to respectively TCC and analysis pipelines?
- Methods:
  - It needs to be described in detail how the fragment length and standard deviation were estimated from the Bottomly data since it is single end data. The actual values should also be reported for reproducibility.
  - Is there a particular reason why Salmon/Kallisto was not run with the bias correction algorithms?
  - Since the authors have to rerun salmon anyway (see major comments) it might be beneficial to update to Salmon v0.13.1 and also use the “--validateMappings” option.
  - Please state the parameters used with Trimmomatic for reproducibility.
  - Please provide info on how the transcript-level counts was obtained (and specify if any scaling was done with e.g. tximport).
  - Please also indicate how the exon/transcript level analysis was summarized to gene-level for each of the 3 workflows.
  - Please provide the unfiltered salmon quantification results (the “quant.sf” files) from the Bottomly *et al* data as supplementary files to facilitate reproducibility.
  - Please provide details of how the STAR mapped data was converted to DEXSeq ready counts (currently only implied in the result section).
- Results:
  - From the first paragraph in results it is not clear that you are actually doing all 3 types of analysis and comparing them. And starting with mentioning the “alternative approach” is not reader friendly.
  - For references to previous DTU benchmarking please also cite Love et al 2018 ^1^.
  - For the “Fewer equivalence classes are expressed than exons” analysis it is unclear whether it is the number of exons or disjointed exon bins necessary for a standard DEXSeq workflow (due to alternative 3’ and 5’ splice sites) which are quantified.
  - Figure 2: Was any pseudocounts or transformation used to calculate the normalized cross replicate variance (Log2(var / mean))?
  - Figure 3: Please add “A” and “B” to the figure in accordance with the figure legend.
  - Figure 3A:
    - What is visualized is not explained in figure legend.
    - It is currently not possible to distinguish between the different methods on the plot. Please provide zoom in versions of the plot to enhance visual comparison.
    - From the point of this paper (comparing the 3 workflows depicted in the lower half of figure 1) it is very strange that multiple exon-based workflows as well as the result of a MISO based workflow (which is never discussed) are also shown. Would it not make more sense to only show exon-based workflow used and omit the MISO based workflow? Furthermore since the supplementary figures show that Salmon and Kallisto produce the same results why not only show one of them?
  - Figure 3B:
    - Please report which FDR cutoff was used to call significance.
    - Please also report the result analysis on the feature level (transcript/exon) and not just for the gene-level.
    - The authors should discuss the much larger variance in FDR for TCC and exon based approaches as well as the generally large FDR values (gussing the target value was 0.05)
- Discussion:
  - “We propose that equivalence classes are the optimal unit for performing count based differential testing” is to broad a claim since this article is about DTU analysis. Save it for a review :-).
  - Please refer to sashimi plots in addition to superTranscripts – they had the visualization idea first.

I have read this submission. I believe that I have an appropriate level of expertise to confirm that it is of an acceptable scientific standard, however I have significant reservations, as outlined above.

## References

[1] LoveMISonesonCPatroR: Swimming downstream: statistical analysis of differential transcript usage following Salmon quantification. *F1000Res*.2018;7: 10.12688/f1000research.15398.3 952 10.12688/f1000research.15398.3 30356428PMC6178912

[2] YiLLiuLMelstedPPachterL: A direct comparison of genome alignment and transcriptome pseudoalignment. *bioRxiv*.2018; 10.1101/444620 10.1101/444620

[3] NtranosVKamathGZhangJPachterLTseD: Fast and accurate single-cell RNA-seq analysis by clustering of transcript-compatibility counts. *Genome Biology*.2016;17(1) : 10.1186/s13059-016-0970-8 10.1186/s13059-016-0970-8 27230763PMC4881296

[4] NtranosVYiLMelstedPPachterL: A discriminative learning approach to differential expression analysis for single-cell RNA-seq. *Nat Methods*.2019;16(2) : 10.1038/s41592-018-0303-9 163-166 10.1038/s41592-018-0303-9 30664774

# Response

**Published:** 2019-04-19

**Authors:** Alicia Oshlack

**Affiliations:** Murdoch Childrens Research Institute, Australia

Thank you for taking the time to review our paper and for the helpful suggestions.

Major comments:

- We ran our EC-based method, as well as the transcript and exon-based methods, through DTU analysis on the simulated data from Love *et al.*[1]. EC-based results can be seen to be on par with transcript-based results (Supplementary Figure 8). As we note in the revised paper, the simulations were based on baseline abundances derived using Salmon, which may have favoured Salmon-derived transcript quantifications in the downstream analysis. Together with the Soneson simulation, this indicates that ECCs perform as well as the best method regardless of the assumptions and biases in the simulated datasets. 
- Regarding filtering, we note that DEXSeq automatically filters out zero-count features and low-count data. In order to show the effects of basic filtering on the EC, transcript and exon-based approaches, we present Supplementary Figure 6. This figure shows that filtering performs slightly better in controlling FDR per approach. Importantly, ECC-based DTU still out-performs transcript-based DTU in both the drosophila and human data.
- We agree with the reviewer’s comment that the pseudo-alignment index should be built on all transcripts and only subsequently filtered. The Soneson simulation data, however, is restricted to protein-coding genes only. All downstream results obtained from the Soneson data were run on references containing protein-coding genes only, therefore we opted to keep references consistent with the EC-based approach for optimal fairness. As we also compared the Bottomly data with the Soneson data in Figure 2, we opted to take the same approach with the Bottomly data. We have noted this decision in the methods. We used the whole transcript index without gene filtering for the Love data.
- We have updated the manuscript for conciseness, and updated labels figures and captions to improve clarity.

Minor comments:

- General, title and abstract:
  - We have opted to retain the use of ‘equivalence class counts’, noting that both ‘transcript compatibility counts’ and ‘equivalence class counts’ are used in the literature.
  - We have updated the manuscript title for clarity.
  - We have addressed the points regarding the abstract.
- Introduction:
  - We have now cited transcript-assembly and spliced-in DTU approaches.
  - We now discuss some of the limitations of pseudo-alignment in the Discussion.
  - We opted to show four equivalence classes in Figure 1 for simplicity. We have noted the possibility of ECs containing solely t2 and t3 in the main text.
  - We have added a reference for the term ‘transcript compatibility counts’.
  - We have corrected the discussion on ideas presented in Ntranos *et al*.[2]
  - We have added (a) and (b) labels for Figures 1-3
- Methods:
  - Fragment lengths and standard deviations were estimated directly from the read lengths (as these varied between reads due to trimming). The length and standard deviation values have been added to the methods section.
  - Salmon/Kallisto were run with default arguments (apart from returning equivalence class counts) in order to run the software more-or-less ‘out of the box’ without parameter tuning, which may take focus away from the conceptual advance of using equivalence classes. Additionally, --validateMappings can be seen as a further optimisation to EC-derivation.
  - Trimmomatic parameters have been added to the methods section. STAR was run with default parameters, which has also been added to the methods section.
  - Tximport with ‘scaledTPM’ scaling was used to obtain transcript abundances from Salmon. This is now reflected in the methods.
  - DEXSeq’s *perGeneQValue *function was used to obtain gene-level significance values. This is now reflected in the methods.
  - Salmon’s “quant.sf” files are available in the ec-dtu-paper github repository. This is now reflected in the methods.
  - We have clarified how exon counts are obtained from STAR counts.
- Results:
  - We have revised the first paragraph for clarity.
  - We now cite Love *et al.*[1] in reference to DTU method benchmarking.
  - The “Fewer equivalence classes are expressed than exons” analysis considers exon counting bins. This has now been clarified in the main text.
  - Cross-replicate log2(var / mean) calculations were performed on CPM-transformed and lightly filtered data. This is now reflected in the methods section.
  - Figure 3
    - We have described the figure in greater detail in the caption.
    - Supplementary Figure 6 has been added, which uses zoomed-in axes and shows results for ECC, exon and transcript counts.
    - We show MISO as the way the method was used in Soneson *et al.* is conceptually similar and have removed feaureCounts and kallisto results to remove clutter.
    - FDR cutoff is now stated in the figure legend.
    - Reporting the results on the feature level is not feasible as truth data is not available at the feature level. Additionally, equivalence classes do not map cleanly to features, which would make it difficult to asses the truth of features even if exon and transcript-level truth were available.
    - For the Bottomly replication data, we now note the FDR variance of the ECC and exon-count based methods, indicating that this may be the result of substructure in the data. Importantly, FDR is lower in all iterations but one for ECCs compared with transcript counts.
- Discussion:
  - We have addressed the suggestions for the discussion.

 

References:

[1] Love, M. I., Soneson, C., & Patro, R. (2018). Swimming downstream: statistical analysis of differential transcript usage following Salmon quantification. *F1000Research*, *7*, 952. https://doi.org/10.12688/f1000research.15398.1

[2] Ntranos, V., Yi, L., Melsted, P., & Pachter, L. (2019). A discriminative learning approach to differential expression analysis for single-cell RNA-seq. *Nature Methods*, *16*(February), 1. https://doi.org/10.1038/s41592-018-0303-9
